# Molecular and cellular pathogenesis of adamantinomatous craniopharyngioma

**DOI:** 10.1111/nan.12226

**Published:** 2015-04-23

**Authors:** Juan Pedro Martinez‐Barbera

**Affiliations:** ^1^ Birth Defects Research CentreDevelopmental Biology and Cancer ProgrammeInstitute of Child HealthUniversity College LondonLondonUK

**Keywords:** adamantinomatous craniopharyngioma, pituitary, Sox2, stem cells, WNT pathway, β‐catenin

## Abstract

Adamantinomatous craniopharyngiomas (ACPs) are the most common pituitary tumours in children. Although histologically benign, these are clinically aggressive tumours, difficult to manage and associated with poor quality of life for the patients. Several human and mouse studies have provided unequivocal evidence that the over‐activation of the WNT/β‐catenin signalling pathway underlies the molecular aetiology of these tumours. Recently, research using genetically modified mouse models of human ACP have revealed a critical and unexpected non‐cell autonomous role for pituitary stem cells in ACP tumourigenesis, which has expanded the cancer stem cell paradigm. As the result of this basic research, the pathogenesis of ACP is being unveiled, with promising implications for the development of novel treatments against these childhood neoplasms. These benign tumours may additionally represent a unique model to provide insights into the initial steps of oncogenesis.

## Introduction

Adamantinomatous craniopharyngiomas (ACPs) are non‐hormone secreting sellar tumours that mostly affect children under 15, but can be diagnosed at any age, with a second peak of incidence in adults between 50 and 74 years. The clinical relevance of ACP is not their associated mortality, which is usually low when properly managed, but their high morbidity due to serious endocrinological disturbances and tendency to infiltrate locally and aggressively into the hypothalamus and visual tracts. Currently, there are no specific treatments against these tumours, only surgery and/or radiotherapy, but these are not curative and patients often experience tumour recurrence. Consequences of the tumour and its treatment are long lasting and associated with poor quality of life for the survivors, making many clinicians consider human ACP as a chronic disease [1, 2].

This review aims to discuss the pathogenesis of human craniopharyngioma from an embryological perspective, arguing that the majority of human ACPs are developmental tumours. It will present results obtained from human and mouse studies, highlighting the implications of data derived from current research using validated mouse models for these human neoplasms. In particular, it will provide an overview on the critical findings that have helped delineate the early cellular and molecular mechanisms resulting in development of ACP, with a particular emphasis on the role of pituitary stem cells. For in‐depth analysis of the clinical aspects of human ACP, such as treatments and patient management, readers are directed to the other reviews on these topics [1, 3, 4, 5, 6, 7, 8, 9, 10, 11, 12].

## Pathology of human ACP

Human ACPs are cystic tumours located either within or above the sellar region, frequently compromising the hypothalamus, pituitary gland, pituitary stalk and optic chiasm. The histopathology of these tumours is characterized by the formation of a peripheral basal cell layer of palisading epithelium, loose aggregates of epithelial stellate cells, nodules of anucleated ghost cells with brightly eosinophilic cytoplasm termed wet keratin, large areas of regressive changes, that is, hemosiderin deposits, cholesterol clefts, multinucleated foreign‐body giant cells, inflammation and calcifications [13, 14, 15, 16].

**Figure 1:**
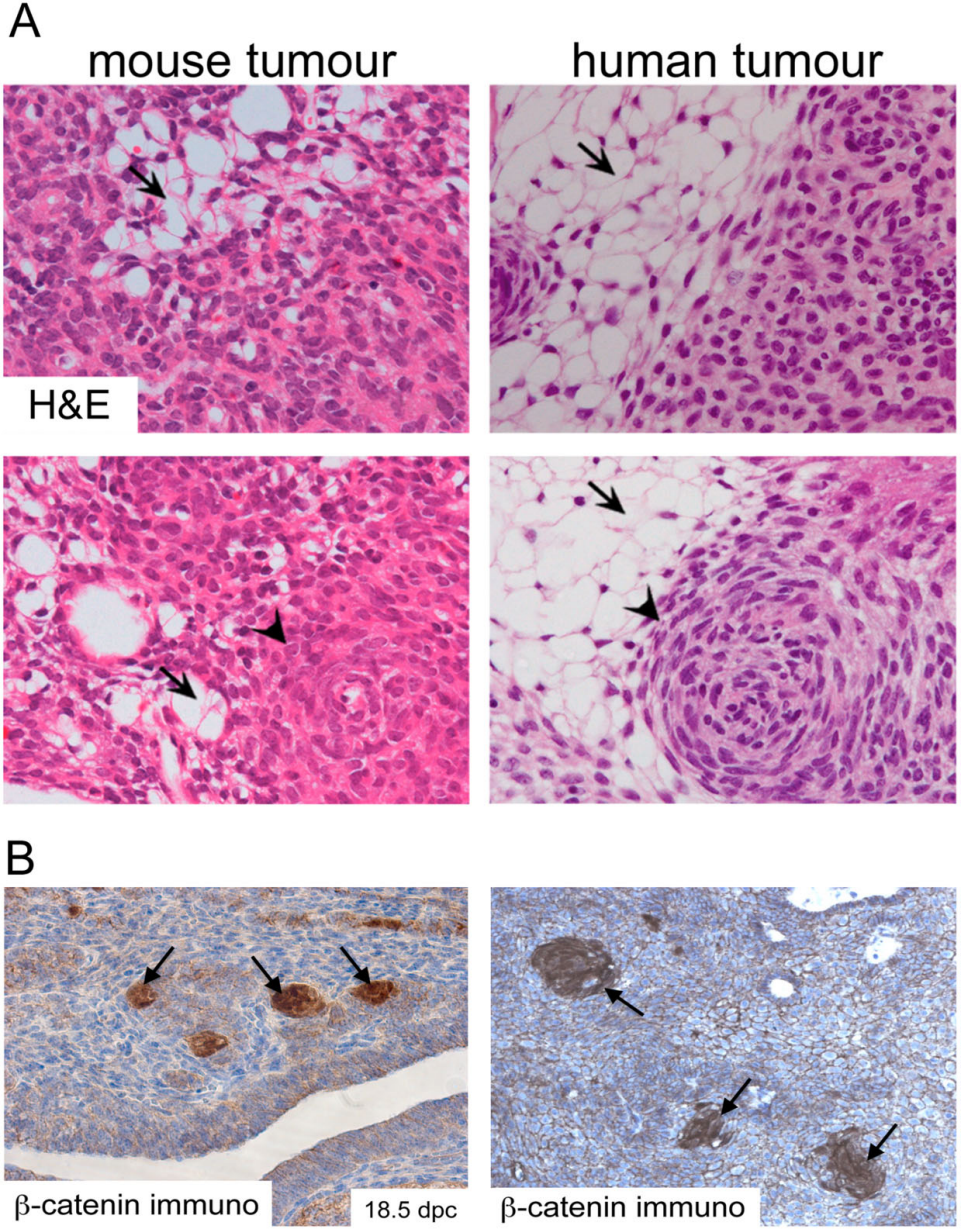
Histomorphological features of mouse and human ACP. (**A**) Haematoxylin and eosin staining of mouse and human tumours showing the presence of microcystic changes (stellate reticulum; arrows) and whorl‐like nodular structures (cell clusters; arrowheads). (**B**) Immunohistochemistry with a specific anti‐β‐catenin antibody showing the presence of cell clusters with nucleo‐cytoplasmic accumulation of β‐catenin. Reproduced with permission from PNAS (Gaston‐Massuet *et al*., PNAS USA 2011, vol. 108, number 28, pp 11482–11487).

A histopathological hallmark of human ACP is the presence of cells showing nucleo‐cytoplasmic accumulation of β‐catenin, which often gather to form whorl‐like structures close to the invasive front (hereby referred to as ‘cell clusters’) [9, 17, 18, 19, 20] (Figure 1). These are clearly a minority of cells and are distributed evenly throughout the tumour. These β‐catenin‐accumulating cluster cells show activation of the WNT/β‐catenin pathway as evidenced by the expression of pathway targets such as *AXIN2*, *LEF1* and *BMP4* [18, 21, 22]. They distinctly express stem cell markers [23] and the tight junction protein Claudin‐1 [24]. The presence of these clusters is a hallmark that differentiates human ACP from any other tumour of the sellar region, including the papillary type of craniopharyngioma [25].

The cell of origin of human ACP remains unknown. The pattern of expression of cytokeratins is consistent with oral epithelial origin, and it is generally believed that these neoplasms derive from remnants of Rathke's pouch, the embryological primordium of the anterior pituitary [26, 27].

## WNT/β‐catenin signalling is required for normal development of the pituitary gland

**Figure 2:**
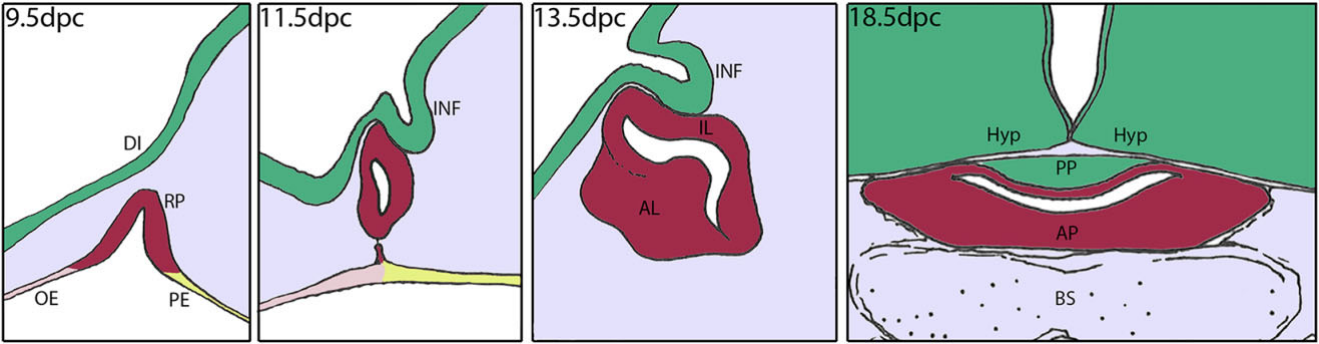
Cartoon showing mouse pituitary development from 9.5 to 18. 5 dpc. Rathke's pouch (RP) forms at 9.5 dpc as an evagination of the oral ectoderm (OE) at the boundary between OE and pharyngeal endoderm (PE). The epithelium of RP contains the undifferentiated progenitors that give rise to all hormone‐producing cells at the end of gestation. By 11.5 dpc, RP pinches off from the oral ectoderm and establishes contacts with the infundibulum (INF), a recess of the floor of the diencephalon (DI). RP progenitors proliferate rapidly from 9.5 dpc and the progeny migrates ventrally and initiates differentiation to populate the primordium of the anterior lobe (AL) of the pituitary. The presumptive intermediate lobe (IL) is indicated. At 18.5 dpc, just prior to birth, the pituitary comprises the posterior lobe (PP), of neural origin, and the anterior pituitary (AP), derived from the anterior and intermediate lobes. The pituitary is located between the hypothalamus (Hyp) and the basosphenoid bone (BS). (Figure courtesy of Dr C. L. Andoniadou.)

The pituitary gland is a master endocrine organ that regulates essential physiological processes including growth, reproduction, metabolism and stress response. The mature gland, consisting of the anterior and posterior pituitary (AP and PP, respectively), starts to develop around 9.0 days post coitum (dpc) in mice and around week 4 in humans from two distinct germ layers (Figure 2). The AP derives from Rathke's pouch, a region of the oral epithelium of the early embryo that contains the undifferentiated precursors of all of the hormone‐producing endocrine cells of the mature pituitary as well as tissue‐specific pituitary stem cells of the adult organ [28]. The PP is of neural origin and originates from a diverticulum at the floor of the prospective hypothalamus called the infundibulum. The PP is hypocellular and contains the axons of the oxytocin and arginine vasopressin neurons of the hypothalamus. The PP is connected to the hypothalamus through the pituitary stalk, which comprises the portal vessels and hypothalamic axons.

**Figure 3:**
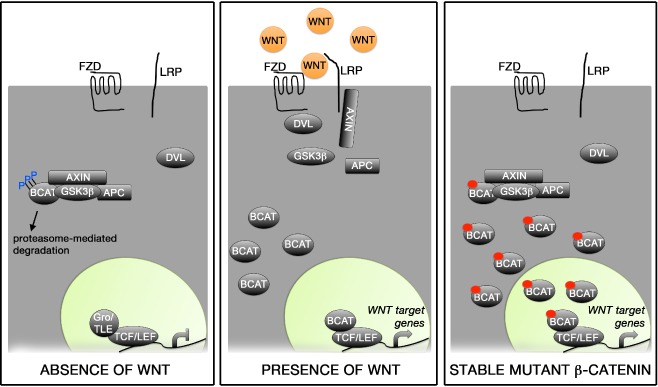
WNT/β‐catenin signalling pathway. (**A**) In the absence of WNT ligands, β‐catenin is trapped in a destruction complex formed by Axin, APC and GSK3β and phosphorylated in specific residues encoded by exon 3 of CTNNB1. This results in rapid degradation of β‐catenin and, consequentially, the repression of target genes brought about by the interaction of TCF/LEF1 with co‐repressors [e.g. groucho (Gro)/TLE family members]. (**B**) Binding of WNT ligands to the Frizzled (FZD) receptors and ist co‐receptors LRP leads to the activation of dishevelled (DVL) and recruitment to the cytoplasmic membrane of both DVL and Axin. This causes the dysruption of the destruction complex, releasing β‐catenin, which cannot be efficiently phosphorylated or degraded. The stabilisation of β‐catenin results in increased cytoplasmic protein levels and eventually nuclear translocation, where its interaction with TCF/LEF1 factors mediates the expression of target genes oft he pathway. (**C**) Expression of a stable form of β‐catenin lacking the amino acids encoded by exon 3 of CTNNB1 (small red circle in BCAT) results in protein stabilisation as mutant β‐catenin cannot be properly phosphorylated or degraded. This causes the activation of the WNT/β‐catenin pathway in a cell‐autonomous manner in the absence of WNT ligands. (Figure courtesy of Dr C. L. Andoniadou.)

Normal morphogenesis and differentiation of the pituitary gland are regulated by the activity of several transcriptional factors and signalling pathways [29, 30, 31]. One of these pathways is WNT/β‐catenin, a major regulator of several cellular processes during organogenesis and adulthood [32, 33]. The activity of the WNT/β‐catenin pathway is controlled by the stability of β‐catenin, a transcriptional activator lacking a DNA binding domain. In the absence of WNT ligands, β‐catenin is phosphorylated by a destruction complex and degraded rapidly, via the ubiquitination pathway, preventing its stabilization and the activation of the pathway. Binding of WNT ligands to their receptors disrupts the destruction complex, preventing the phosphorylation of β‐catenin and its subsequent ubiquitination and degradation. Stabilized β‐catenin can translocate into the nucleus and interact with DNA‐binding transcription factors of the TCF/LEF family to activate the expression of target genes of the pathway (Figure 3).

Genetic studies in mice have demonstrated the requirement of this pathway for normal pituitary morphogenesis and differentiation. WNT/β‐catenin is necessary for terminal differentiation of hormone‐producing cells at late stages of pituitary development, while it must be inhibited at early stages to establish normal proliferation of Rathke's pouch progenitors [34, 35, 36, 37, 38, 39]. Supporting this notion, the up‐regulation of the WNT/β‐catenin pathway in Rathke's pouch (RP) progenitors leads to dramatic pituitary hyperplasia, ectopic pituitary tissue in the oropharyngeal cavity and perinatal death in 80% of pups. In addition, somatotroph, lactotroph and thyrotroph differentiation is impaired [21]. This genetically modified mouse model harbours a mutated form of β‐catenin that is transcriptionally active, but lacks the amino acids encoded by exon 3 of *Ctnnb1* (the gene encoding β‐catenin), preventing its phosphorylation and subsequent degradation, and leading to the over‐activation of the WNT/β‐catenin pathway [40].

## Over‐activation of the WNT/β‐catenin pathway underlies the molecular aetiology of human and mouse ACP

**Figure 4:**
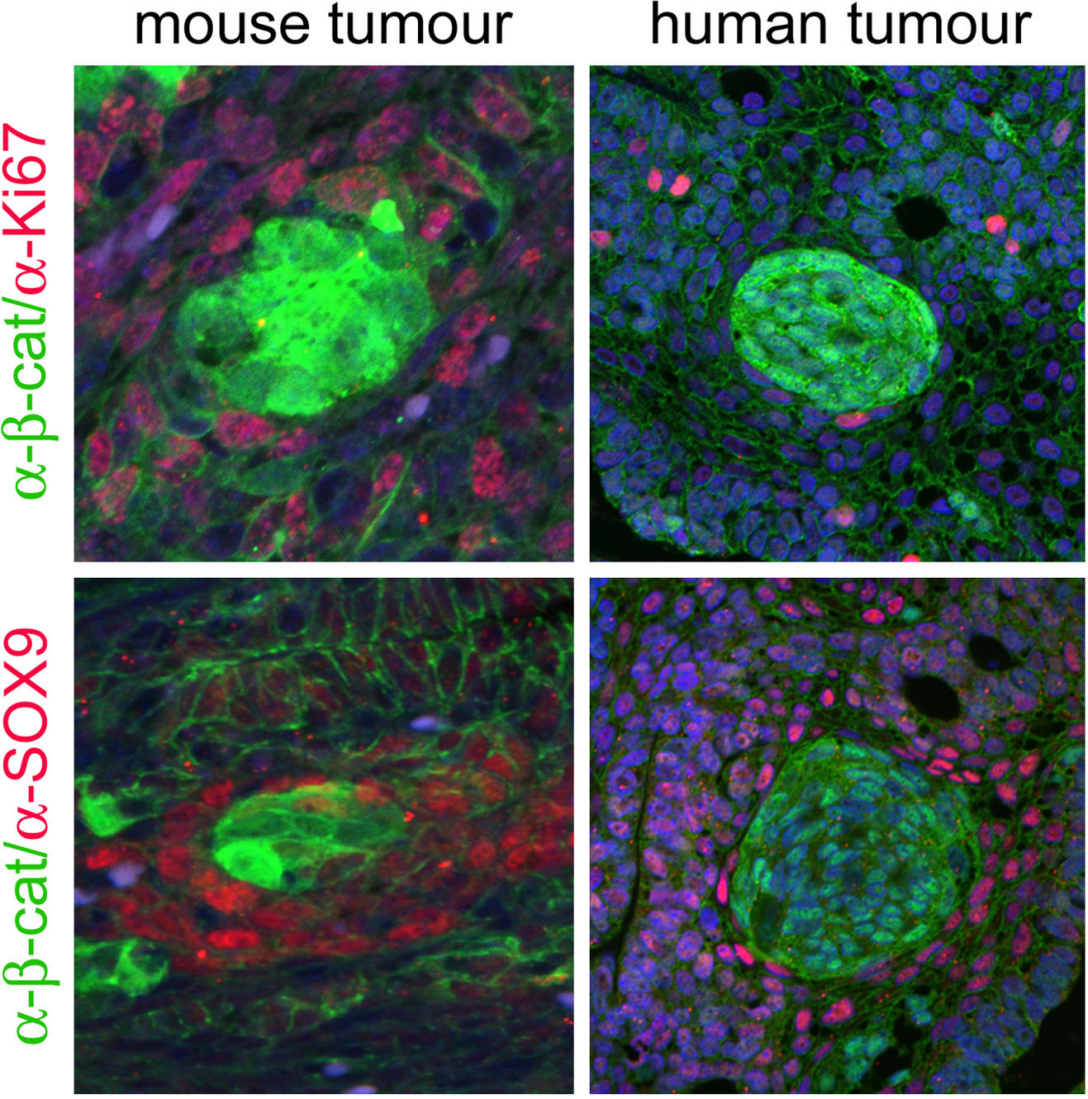
Cluster cells in mouse and human ACP do not express Ki67 or SOX9. Double immunofluorescent staining against β‐catenin (green) and the proliferative marker Ki67 or the transcription factor SOX9 (red). Reproduced with permission from PNAS (Gaston‐Massuet *et al*., PNAS USA 2011, vol. 108, number 28, pp 11482–11487).

An interesting observation of the previously described embryonic mouse line (hereby referred to as the ‘embryonic ACP model’) is that the remaining 20% of the pups survive weaning but show variable growth defects and fully penetrant pituitary tumours, with a median survival of around 11 weeks of age [21]. Histopathological analyses have revealed characteristic hallmarks of human ACP such as large cysts, cell clusters with nucleo‐cytoplasmic accumulation of β‐catenin and expression of *Axin2*, *Lef1* and *Bmp4*, indicating the activation of the Wnt/β‐catenin pathway [21, 25, 41]. These neoplastic cells do not express markers of proliferation or pituitary hormones and are negative for synaptophysin, a marker of neurons and endocrine cells (Figure 4 and data not shown) [21, 42, 43]. Therefore, cluster cells in mouse and human ACPs have exited the cell cycle, are undifferentiated and possibly not of either neural or endocrine origin. However, contrary to the human counterpart, mouse ACPs do not show wet keratin or calcification, suggesting species‐specific differences between mouse and human ACPs. Critically, human ACPs develop over years, but mouse ACPs develop in a period of weeks. Despite these differences, the mouse tumours are more similar to human ACP than to any other tumour of the sellar region including pituitary adenoma, Rathke's cleft cysts and even the papillary type of craniopharyngioma.

Mutations in *CTNNB1* have been identified in most of the human ACP samples analysed [17, 19, 20, 44, 45, 46]. These are mostly restricted to exon 3 and result in the substitution or deletion of the critical regulatory amino acids that control stabilization of the protein, predicted to lead to its nucleocytoplasmic accumulation and over‐activation of the WNT/β‐catenin pathway [21, 22, 25, 41, 47]. Human mutations are functionally equivalent to the mutated β‐catenin expressed in the embryonic ACP model. Together, the human and mouse studies have provided robust evidence for a role of *CTNNB1* mutations and the over‐activation of the WNT/β‐catenin pathway in the molecular aetiology of ACP.

## Childhood‐onset ACP is likely to be a tumour of embryonic origin

Further molecular and genetic studies using the embryonic ACP mouse model have revealed two interesting findings on the pathogenesis of human ACPs, in particular, of childhood‐onset ACP. First, genetic approaches in mice have demonstrated that tumours form only when oncogenic β‐catenin is expressed in Rathke's pouch undifferentiated precursors, but not when committed progenitors or fully differentiated hormone‐producing cells are targeted. Second, analyses of mouse embryos at different stages of development have revealed that β‐catenin‐accumulating cell clusters with a similar gene expression profile to the human clusters are present in the pituitary gland of the embryonic ACP model by the end of gestation [21].

Are these conclusions applicable to the human tumours? The similarities between the mouse and human clusters are remarkable, not only morphologically but also at the molecular level, strongly suggesting that they represent equivalent structures in mouse and human ACPs. In fact, whole transcriptome gene expression profiling of the mouse cluster cells has revealed numerous genes/pathways that are dysregulated in the mouse and also in the human tumours [42]. The possibility that ACP patients may have cell clusters in the pituitary gland is supported by clinical evidence. In a retrospective analysis of 90 children diagnosed with ACP (median age at diagnosis 8.3 years), Muller and colleagues have reported a significant decrease in height score at 10–12 months of age that persisted until the diagnosis of ACP [48]. Of note, somatotrophs are clearly defective in the embryonic ACP model by the end of gestation and postnatally resulting in dwarfism [21]. Therefore, it is tempting to suggest that clusters may develop embryologically in human patients and may be present in the pituitary gland of the newborn prior to the appearance of any tumour mass. In fact, foetal ACP was recently diagnosed in a 18 week human foetus [49].

## Pituitary stem cells play a critical role in the pathogenesis of ACP

The presence of tissue‐specific stem cells has been demonstrated in several organs including the brain, gastrointestinal tract, skin, bone marrow and skeletal muscle among others. These cells can self‐renew, express markers associated with stemness (e.g. *Sox2*) and can give rise to progeny able to differentiate and contribute to tissue homeostasis as well as regeneration [32, 50]. The existence of similar cells in the adult murine pituitary gland has been long suspected [51], but not fully demonstrated until recently, by using genetic tracing in the mouse [52, 53]. Pituitary stem cells express the transcription factors SOX2 and SOX9 and are capable of differentiating into all of the hormone‐producing cells to contribute to organ homeostasis and regeneration. In the adult human pituitary, SOX2+ve cells have also been identified, suggesting that stem cells may also exist in the human organ [54].

Tissue‐specific stem cells can be the origin of cancer, a concept that has fuelled the cancer stem cell (CSC) paradigm. This proposes that within tumours and cancers, there is a minority of transformed cells capable of self‐renewing and generate the progeny that populate the tumour bulk. As CSCs may be particularly resistant to chemotherapy and radiation, it is thought that they can be responsible for cancer evolution, recurrence and metastasis, raising a significant interest in the oncology field as potential targets for novel specific therapies [55, 56, 57, 58, 59, 60].

The presence of CSCs in pituitary tumours, and specifically in human ACP, has recently been investigated using genetic approaches. A mouse model was generated by activating the WNT/β‐catenin pathway exclusively in SOX2+ve adult pituitary stem cells [52]. These mice, referred to as the ‘adult ACP model’, also develop pituitary tumours that are composed of synaptophysin‐negative and undifferentiated (i.e. neither endocrine nor neural) tumour cells. Importantly, these tumours contain β‐catenin‐accumulating cell clusters, indicating a significant resemblance to both human ACP and the embryonic model previously described. Taking into consideration this mouse research, it could be speculated that mutations in *CTNNB1* in SOX2+ve stem cells of the mature adult pituitary are the cause of adulthood‐onset ACP in humans, while the same mutations in undifferentiated RP progenitors may lead to childhood‐onset ACP. Further molecular characterization of adult and childhood ACP may help resolve this hypothesis.

**Figure 5:**
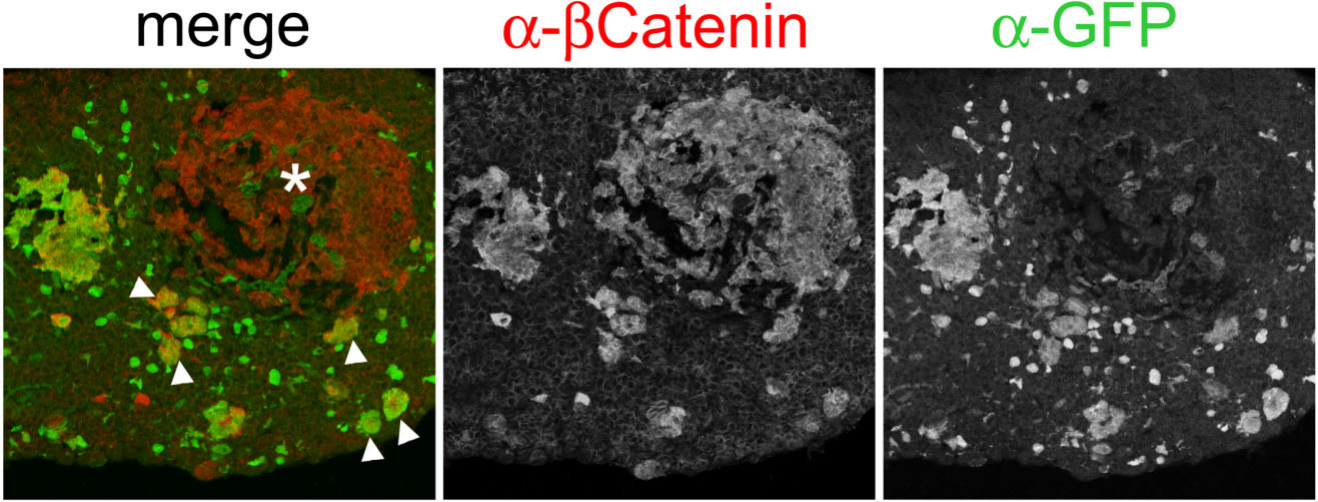
The progeny of SOX2^+^ pituitary stem cells give rise to the cell clusters but are not the cell‐of‐origin of mouse ACP. Double immunofluorescence staining reveals the presence of a β‐catenin‐accumulating tumour (asterisk) that is YFP ^−ve^. Note that YFP ^−ve^ tumour is adjacent to YFP ^+ve^ cell clusters showing nucleo‐cytoplasmic β‐catenin accumulation (arrowheads). Reproduced with permission from Elsevier (Andoniadou *et al*., Cell Stem Cell 2013, vol. 13, issue 4, pp 433–445).

It was important to address whether the mutated SOX2+ve cells in the adult ACP model were CSCs. To address this important question, SOX2+ve stem cells have been targeted to simultaneously express oncogenic mutant β‐catenin and yellow fluorescent protein (YFP). If normal SOX2+ve cells were transformed into CSCs due to the expression of oncogenic β‐catenin, the tumour would be expected to contain YFP+ progeny cells. Surprisingly, tumours do not express YFP, indicating that the cell of origin of mouse ACP is not a targeted SOX2+ve cell (Figure 5) [52]. The targeted SOX2+ve cells proliferate transiently due to the over‐activation of the WNT/β‐catenin pathway; but soon after this burst of cell division, they exit the cell cycle and form cell clusters with nucleo‐cytoplasmic accumulation of β‐catenin, like those observed in human ACP. Two conclusions can be drawn from these studies: (i) SOX2+ve stem cells are not the cell of origin of ACP; however, they are the origin of the typical cell clusters that characterize these neoplasms; (ii) mutated SOX2+ve cells expressing oncogenic β‐catenin are not CSCs according to the classical definition of the CSC paradigm, since their descendants do not colonize the tumours, rather they induced tumours in a non‐cell autonomous manner.

## A paracrine model for the involvement of pituitary stem cells in tumourigenesis

If murine ACP tumours are not derived from the mutated SOX2+ve stem cells in a cell‐autonomous manner, what is the mechanism underlying tumour formation? Molecular analyses have provided some interesting and provocative insights into this question. Using genetic approaches, the cluster cells have been isolated from the embryonic mouse model and their gene expression profiled in comparison with the non‐cluster tumour cells to identify dysregulated genes/pathways [42]. The studies have revealed that cluster cells function like signalling centres by expressing a great variety of secreted proteins including members of the fibroblast growth factor (FGF), transforming growth factor beta (TGFβ), epithelial growth factor (EGF) and sonic hedgehog (SHH) pathways. In addition, several chemokines and cytokines as well as their receptors, which are critical mediators of inflammation, are also up‐regulated in the mouse clusters. Of interest, proteins with pro‐inflammatory properties have been identified in the cystic fluid of human ACP, suggesting that ACP may be an inflammation‐driven tumour [13, 14, 15, 16]. Many of these factors have been shown to be expressed in several tumours/cancers and play critical roles in tumour biology [61, 62, 63, 64, 65].

**Figure 6:**
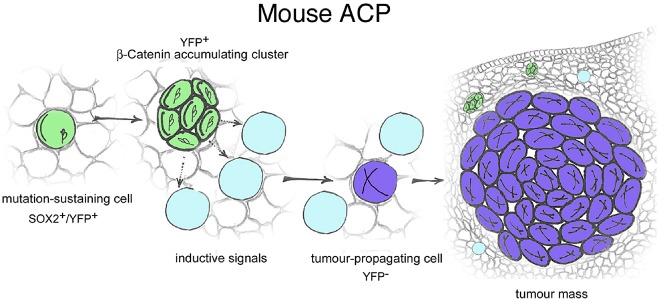
Paracrine model of involvement of pituitary stem cells in tumourigenesis. When targeted to express oncogenic β‐catenin, SOX2^+ve^ cells (green) accumulate nucleo‐cytoplasmic β‐catenin, proliferate transiently and stop dividing forming clusters. Clusters secrete signals to the surrounding cells to induce cell transformation and tumour growth from a cell that is not derived from the targeted SOX2^+ve^ stem cell. Reproduced with permission from Elsevier (Andoniadou *et al*., Cell Stem Cell 2013, vol. 13, issue 4, pp 433–445).

Normal embryogenesis relies heavily on signalling centres. Broadly speaking, normal organogenesis including pituitary morphogenesis is brought about by the activities of cells with instructive properties that secrete signals (i.e. signalling centres) and those of surrounding cells, which respond to those signals. In mouse ACP, β‐catenin‐accumulating cell clusters secrete factors affecting the tissue microenvironment in a paracrine manner, promoting cell transformation and tumourigenesis. In this paracrine model of involvement of stem cells in tumourigenesis, the cell sustaining the oncogenic mutation and the cell of origin of the tumours are different [52] (Figure 6).

An important question is to what extent these findings are relevant to human ACP. Several lines of evidence support the view that the pathogenesis of mouse and human ACP are very similar: (i) recent research using a xenograft model of human ACP in immunosuppressed mice has shown that the activities of the clusters are critical to regulate cell behaviour in the invasive front of the tumours [66]; (ii) human clusters also express FGFs, TGFβs, SHH, chemokine receptors and other secreted factors capable of modifying the tumour microenvironment and promoting tumour growth [22, 42, 67, 68]; (iii) cluster cells in the human tumours are non‐proliferative, undifferentiated (i.e. neither endocrine not neural) and express markers associated with stem cells [21, 23]; (iv) cell clusters are present in the majority of human ACP, even in very large and advanced tumours, suggesting that their activities must be required for growth and/or survival of tumour cells. If this was not the case, it would be difficult to explain why the tumour ends up killing all of the normal pituitary tissue, but sparing a limited population of non‐dividing cells forming clusters. Whether the paracrine signals of the clusters may also be required for the initial transformation of the cell of origin of the tumour is an important question that requires further research. Moreover, it is possible that the paracrine model may be applicable to other pituitary tumours and human cancers as published evidence suggests its broader implications in the oncology field [69, 70, 71, 72, 73, 74].

## Conclusions and open questions

The pathogenesis of human ACP is being revealed and several dysregulated genes and pathways have been identified, which may impact the diagnosis, prognosis and treatment of patients in the years to come. It is established that mutations in *CTNNB1* resulting in the over‐activation of the WNT/β‐catenin pathway underlie the aetiology of ACP, but many questions remain unanswered. Why are the tumourigenic effects of mutant β‐catenin restricted only to Rathke's pouch embryonic precursors and adult pituitary stem cells? Research from mouse models has demonstrated that only these undifferentiated cells are able to induce tumours when targeted with oncogenicβ‐catenin. This is likely to be the case in human ACP, when taking into account the similarities in the pathogenesis and the possibility that childhood‐onset ACP is likely to be a developmental tumour.

Which is the cell of origin of the tumours? It is puzzling that mutated SOX2+ve stem cells in the adult pituitary are not the cell of origin of the tumours in the mouse as it would be expected according to the CSC paradigm. Instead they induce tumours in a non‐cell autonomous manner by activating a secretory phenotype with tumour‐inducing potential. Whether cluster cells may or may be not the origin of human ACP requires detailed molecular analyses. Of note, Holsken *et al*. (2009) revealed the presence of different *CTNNB1* mutations inside and outside the clusters in 50% of the human ACP analysed, suggesting the existence of tumour heterogeneity as described in mouse ACP [22].

Which are the dysregulated pathways in human ACP? Mouse and human studies have discovered several pathways that are dysregulated in ACP, but non‐biased large‐scale gene expression analyses of human ACP are needed to identify other targetable pathways and novel biomarkers capable of predicting tumour behaviour such as infiltrative potential and recurrence.

Could the study of these benign tumours provide novel insights into the origin of cancer? If cancer is considered as the malignant progression of a pre‐malignant lesion, then benign tumours offer a window of opportunity to better understand the early steps of cell transformation and tumour initiation. Such knowledge is needed if we aim to improve early diagnosis and to develop treatments, which eliminate tumour cells at more vulnerable stages of cancer progression. The paracrine mechanism described using the adult ACP model has broadened our knowledge on how adult stem cells contribute to tumourigenesis beyond the classical CSC paradigm. Future research is required to dissect this model further and to assess its impact in the cancer field.

## Conflict of interest

The author declares no conflict of interest.
